# Sex and COVID-19 vaccination uptake and intention in the Democratic Republic of Congo, Nigeria, Senegal, and Uganda

**DOI:** 10.3389/fgwh.2024.1356609

**Published:** 2024-06-12

**Authors:** Rawlance Ndejjo, Nuole Chen, Steven N. Kabwama, Eniola A. Bamgboye, Marc Bosonkie, Oumar Bassoum, Suzanne N. Kiwanuka, Mobolaji M. Salawu, Landry Egbende, Ndeye Mareme Sougou, Rotimi F. Afolabi, Mamadou Makhtar Mbacké Leye, Segun Bello, Ayo S. Adebowale, Magbagbeola D. Dairo, Ibrahima Seck, Olufunmilayo I. Fawole, Mala Ali Mapatano, Lily L. Tsai, Rhoda K. Wanyenze

**Affiliations:** ^1^ Department of Disease Control and Environmental Health, School of Public Health, College of Health Sciences, Makerere University, Kampala, Uganda; ^2^ Department of Political Science, Massachusetts Institute of Technology, Cambridge, MA, United States; ^3^ Department of Community Health and Behavioral Sciences, School of Public Health, College of Health Sciences, Makerere University, Kampala, Uganda; ^4^ Department of Epidemiology and Medical Statistics, Faculty of Public Health, College of Medicine, University of Ibadan, Ibadan, Nigeria; ^5^ Department of Nutrition, Kinshasa School of Public Health, School of Medicine, Kinshasa, Democratic Republic of Congo; ^6^ Department of Preventive Medicine and Public Health, Faculty of Medicine, Pharmacy and Odontology, Cheikh Anta Diop, University, Dakar, Senegal; ^7^ Department of Health Policy, Planning and Management, School of Public Health, College of Health Sciences, Makerere University, Kampala, Uganda

**Keywords:** COVID-19, sex, trust, vaccines, vaccination, Africa

## Abstract

The introduction of vaccines marked a game changer in the fight against COVID-19. In sub-Saharan Africa, studies have documented the intention to vaccinate and the uptake of COVID-19 vaccines. However, little is documented about how sex differences could have impacted COVID-19 vaccination. We conducted a multi-country cross-sectional study to assess the sex differences in COVID-19 vaccine uptake and intention to vaccinate in the Democratic Republic of Congo (DRC), Nigeria, Senegal, and Uganda. This study involved analysis of data from mobile surveys conducted between March and June 2022 among nationally constituted samples of adults in each country. Bivariate and multivariable logistic regression models were run. The self-reported uptake of COVID-19 vaccines was not significantly different between males and females (*p *= 0.47), while the intention to vaccinate was significantly higher among males (*p* = 0.008). Among males, obtaining COVID-19 information from health workers, testing for COVID-19, and having high trust in the Ministry of Health were associated with higher vaccination uptake. Among females, having high trust in the government was associated with higher vaccination uptake. For intention to vaccinate, males who resided in semi-urban areas and females who resided in rural areas had significantly higher vaccination intention compared to their counterparts in urban areas. Other factors positively associated with vaccination intention among males were trust in the World Health Organization and perceived truthfulness of institutions, while males from households with a higher socio-economic index and those who had declined a vaccine before had a lower vaccine intention. Overall, the factors differentiating vaccine uptake and intention to vaccinate among males and females were mostly related to trust in government institutions, perceived truthfulness of institutions, and respondent's residence. These factors are key in guiding the tailoring of interventions to increase COVID-19 vaccine uptake in sub-Saharan Africa and similar contexts.

## 1 Introduction

Vaccines remain an important public health intervention to minimize COVID-19 mortality and morbidity. In the first year of the pandemic, COVID-19 vaccines were estimated to have averted more than 19 million excess deaths (1). Yet, during this period, 95% of the global population was yet to receive a single dose of a vaccine and only 1% of the population in Africa had received a vaccine (2). Thus, the number of deaths averted due to vaccination could have been higher (1). Vaccine access and availability during the first year of the pandemic was attributed to a multitude of factors including vaccine nationalism and inequities at global and local levels. Later, the limited manufacturing capacity, vaccine hesitancy, fragile health systems and low investment in vaccine research and development also impacted access and availability of vaccines in Africa (3).

In sub-Saharan Africa, studies have documented differences in willingness and intention to vaccinate against COVID-19 and the uptake of vaccines among various sub-populations (4–6). A systematic review and meta-analysis noted that among the seven million participants in the analysis, women were less likely to accept vaccines compared with men (7). Another scoping review found that age, level of education and gender were significant predictors of vaccination acceptance (5). In light of these findings, researchers have called for an intersectoral gender approach to both the development and deployment of COVID-19 vaccines, highlighting the biological sex differences that affect immune response and the socially constructed differences that influence acceptance, access, and uptake (8). However, little is documented about the impact of sex differences on COVID-19 vaccination acceptance, intention, and uptake.

Emerging evidence from low- and middle-income countries suggests that women may be less likely to trust COVID-19 vaccines and intend to get vaccinated against COVID-19 due to low levels of education, digital gaps, work obligations, and domestic care obligations (9). Similarly, compared with men, women were less likely to receive relevant or trustworthy vaccine information (10). Disparities in access to life-saving public health interventions have significant public health implications and need to be addressed to prevent associated morbidities and mortalities. We conducted a multi-country cross-sectional study that aimed to examine the sex differences in the COVID-19 vaccine uptake and intention in the Democratic Republic of Congo (DRC), Nigeria, Senegal, and Uganda. This information will be useful for the development of gender-specific interventions for the promotion of vaccine acceptance and uptake in public health emergencies.

## 2 Materials and methods

### 2.1 Study area, design and population

This study involved analysis of data from cross-sectional mobile surveys conducted between March and July 2022 in DRC, Nigeria, Senegal, and Uganda among nationally constituted samples of adults. According to the Johns Hopkins University Coronovirus Resource Center, in DRC from March 1–July 31, officially recorded COVID-19 cases since the start of the pandemic rose from 86,039–92,173, while individuals who received at least one dose of a COVID-19 vaccine increased from 754,459–3,450,478. In Nigeria, officially recorded cases rose from 254,570–260,977 and individuals with at least one dose of a COVID-19 vaccine increased from 21,049,754–36,549,506. In Senegal, officially recorded cases rose from 85,699–87,386 and individuals with at least one dose of a COVID-19 vaccine stayed at 1,457,116. And in Uganda, officially recorded cases rose from 163,342–169,230 and individuals with at least one dose of a COVID-19 vaccine rose from 14,247,523–18,081,463. As many people did not have access to testing for COVID-19, these official numbers are likely underreporting the cases during this time.In all countries, at the time of the survey, vaccine availability had increased and vaccination had been opened up to all adults unlike at the start of the vaccination campaign were mostly high-risk groups were targeted.

The determination of the sample size for each country followed quota sampling that reflected the national case distributions of COVID-19 at the time for all countries except Senegal, which used its national census as it did not have the COVID-19 case distributions at the time. Each country stratified the distribution by sex, age, and region (Supplementary Table S1 shows the quotas for each country). World Health Organization (WHO) statistics as of September 2022 indicated COVID-19 vaccination coverage of 5.1% (DRC), 14.7% (Senegal), 30.7% (*N*igeria) and 54.8% (Uganda). Using mobile phones, trained research assistants administered the survey questionnaire following consent from respondents.

### 2.2 Sample size estimation

Each country conducted their own sample size estimation. For the DRC, Senegal, and Uganda, the sample size was determined using the Leslie Kish formula for cross-sectional studies following the assumptions: two-sided Z statistic corresponding to a 95% confidence interval (1.96), 50% as the vaccination adherence level, 5% as the precision level, a design effect of 2.5, and a non-response rate of 10%. In each of these countries, the total sample size estimate was *n* = 1,056. In Nigeria, previous studies had indicated that the willingness of COVID-19 vaccine uptake was 58.2%, resulting in *n* = 1,048.

### 2.3 Sampling strategy

Recruitment into the survey occurred through telephone calls, where each country obtained telephone numbers from first or nongovernmental organisations who had databases of available numbers. Each country used simple random sampling to select phone numbers to call from their database of phone contacts. Each country received phone numbers from all regions in their countries for quota sampling. Not every phone number worked and for numbers that did not work, trained research assistants moved to the next number. Each country had access to additional numbers to ensure that they reached their sample estimates.

### 2.4 Data collection

The survey questionnaire was developed following prior research conducted in Uganda (11). In each country, the study questionnaire was adjusted to fit the context of each country, pretested, and translated into national and major languages in each country. Trained research assistants affiliated with universities in each country conducted telephone interviews in their respective countries. During data collection, high frequency data quality checks and back checking was conducted in each country. Datasets from the four countries were merged and analyzed to answer the research questions. Univariate analysis was conducted, and descriptive statistics provided in the form of means [standard deviation (SD)] for continuous variables and frequencies and proportions for categorical variables.

The survey questionnaire asked respondents about their uptake of COVID-19 vaccines, intention to vaccinate among those who were unvaccinated, and a number of sociodemographic, attitudinal, and other questions related to COVID-19. Independent variables in the study included sociodemographic and economic characteristics, history of adult vaccination, knowledge and information regarding COVID-19 vaccines, source of information on COVID-19, trust in the Ministry of Health and other institutions, among others as detailed elsewhere (11). Indicies for socioeconomic status, trust in academic institutions, trust in government, trust in the Ministry of Health, the how truthful institutions were about COVID-19 were created. Socioeconomic status was generated as an additive index from six variables based on household ownership of television, computer, sofa set, refrigerator, cassette/CD/DVD player, and access to electricity. The socioeconomic index was then categorized into low, middle, and higher, where those who owned 1–2 items were low, 3–4 items were middle, and 5–6 items were higher. All trust-related indicies included several questions related to attitudes about the institutions and indicies were created by taking the mean of those questions. Supplementary Table S2 shows how all indicies are made. The dependent variables were: (1) self-reported uptake of any-approved COVID-19 vaccine and (2) intention to be vaccinated against COVID-19 among those who were unvaccinated. Supplementary Table S2 also shows how each dependent variable was measured in the survey.

### 2.5 Data analysis

To determine the association between sex and uptake of COVID-19 vaccines and intention to vaccinate, a two-step process was used similar to that reported elsewhere (11). First, bivariate analyses were conducted between all variables and the outcomes of interest, uptake of COVID-19 vaccines and intention to vaccinate. Variables where correlations had *p*-values ≤ 0.05 were kept for the multivariable analysis, as well as key sociodemographic variables, age, and education. This two-step process was used for every regression model, including subgroup analyses. Logistic regression models with country-level fixed effects (12) were used for bivariate and multivariable models. Odds ratios (OR) or adjusted odds ratios (AOR) and corresponding 95% Confidence Intervals (CIs) were reported. All analyses were conducted in R V.4.2.2 (R Core Team, 2022).

### 2.6 Ethical considerations

Before commencement of data collection in each of the countries, the study protocols were approved by the national ethics committees. This approval was granted by the Kinshasa School of Public Health Ethics Committee in the DRC, the National Health Research Ethics Committee in Nigeria, the National Committee of Ethics and Research in Senegal, and the Makerere University School of Public Health Research and Ethics Committee in Uganda. Whereas the study protocol was developed in English, it was translated into French in DRC and Senegal to ease data collection and align with country's administrative and cultural requirements.

## 3 Results

### 3.1 Sociodemographic characteristics of respondents

This study involved 4,490 participants, more than half (56.7%) of whom were males. The majority of study participants were aged between 18 and 35 years (51.0%), had attained secondary education as the highest qualification (41.4%), and resided in urban areas (55.7%). Besides occupation, the sociodemographic characteristics were significantly different between males and females (*p *< 0.05) (Table 1). Males and females are significantly different in the study because the COVID-19 case distribution was used to create the samples, and more males had COVID-19 than females. However, the difference in the number of males and females in the study should not affect the results as the estimates are of log odds and odds ratios. In addition, separate analyses for males and females are conducted to understand the relationship of independent variables with dependent variables for each sex.

**Table 1:** Sociodemographic characteristics of respondents.

Characteristic	Total	Male (*n*)	Female (*n*)	*p*-value
	*n* (%)	*n* (%)	*n* (%)	
Overall	4,490 (100)	2,547 (56.7)	1,943 (43.3)	
Age				**<0** **.** **001**
18–35	2,292 (51.0)	1,203 (47.2)	1,089 (56.0)	** **
36–55	1,666 (37.1)	1,022 (40.1)	644 (33.1)	** **
56–65	325 (7.2)	186 (7.3)	139 (7.2)	** **
66+	207 (4.6)	136 (5.3)	71 (3.7)	** **
Country				**<0** **.** **001**
DRC	1,075 (23.9)	643 (25.2)	432 (22.2)	** **
Nigeria	1,148 (25.6)	619 (24.3)	529 (27.2)	** **
Senegal	1,057 (23.5)	557 (21.9)	500 (25.7)	** **
Uganda	1,210 (26.9)	728 (28.6)	482 (24.8)	** **
Education				**<0** **.** **001**
No School/illiterate	214 (4.8)	93 (3.7)	121 (6.2)	** **
Other	137 (3.1)	89 (3.5)	48 (2.5)	** **
Primary	618 (13.8)	344 (13.5)	274 (14.1)	** **
Secondary	1,859 (41.4)	1,034 (40.6)	825 (42.5)	** **
Tertiary/Postgraduate	1,603 (35.7)	963 (37.8)	640 (32.9)	** **
NA	59 (1.3)	24 (0.9)	35 (1.8)	** **
Occupation				**<0** **.** **001**
Unemployed/Retiree/Housewife	783 (17.4)	280 (11.0)	503 (25.9)	
Employed	1,008 (22.4)	695 (27.3)	313 (16.1)	
Self-employed	1,455 (32.4)	861 (33.8)	594 (30.6)	
Casual Laborer	116 (2.6)	84 (3.3)	32 (1.6)	
Farmer	399 (8.9)	265 (10.4)	134 (6.9)	
NA	729 (16.2)	362 (14.2)	367 (18.9)	
Residence				0.16
Urban	2,501 (55.7)	1,392 (54.7)	1,109 (57.1)	
Rural	1,413 (31.5)	831 (32.6)	582 (30.0)	
Semi-urban	560 (12.5)	315 (12.4)	245 (12.6)	
NA	16 (0.4)	9 (0.4)	7 (0.4)	
Socioeconomic index				**0** **.** **02**
Low	1,250 (27.8)	735 (28.9)	515 (26.5)	
Middle	1,781 (39.7)	967 (38.0)	814 (41.9)	
Higher	1,419 (31.6)	828 (32.5)	591 (30.4)	
NA	40 (0.9)	17 (0.7)	23 1.2)	

NA, Not available/missing data.

Bold indicates statistically significant.

### 3.2 Reasons for COVID-19 vaccination uptake and intention by sex

Across the four countries, among both males and females, less than half had been vaccinated with 1,233 (48.5%) males and 928 (47.9%) females reporting that they received at least one dose of the COVID-19 vaccine (see Table 2). The most common reasons for vaccination among both males and females were protection of self and others, a high perceived risk of getting COVID-19 and recommendations by a health worker (See Supplementary Figure S1).

**Table 2:** Association between sex and COVID-19 vaccination intention and uptake.

Variables	Yes *n* (%)	OR (95% CI)	*p*-value	AOR (95% CI)	*p*-value
Vaccine uptake					
Male	1,233 (48.5)				
Female	928 (47.9)	1.02 (0.88,1.18)	0.780	1.08 (0.87,1.34)	0.470
Vaccination intention					
Male	825 (63.4)				
Female	541 (54.0)	0.71 (0.60, 0.85)	<0.001	0.73 (0.58, 0.92)	0.008
Vaccination intention by age					
Males ≤ 40 years	550 (61.6)				
Females ≤ 40 years	420 (53.0)	0.74 (0.60, 0.90)	0.003	0.69 (0.54, 0.89)	0.004
Males > 40 years	275 (67.2)				
Females > 40 years	121 (57.6)	0.69 (0.47, 1.00)	0.047	0.98 (0.55, 1.75)	0.959

Significant variables are highlighted for AOR only.

OR, odds ratio; AOR, adjusted odds ratio.

**Figure 1:**
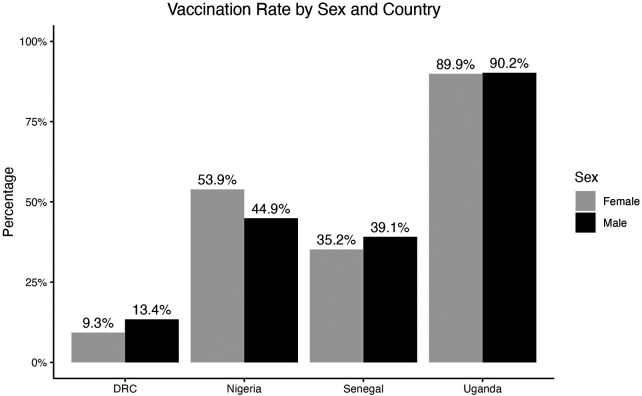
Percentage of respondents who reported that they had received at least one dose of a COVID-19 vaccine, separated by country and sex.

Figure 1 shows the percentage of males and females who reported that they had been vaccinated by country. DRC had lowest vaccination rates, with 9.3% of females and 13.4% of males reporting they had received at least one dose of a COVID-19 vaccine. Uganda had the highest vaccination rates, with 89.9% of females and 90.2% of males reporting that they had received at least one dose of a COVID-19 vaccine. In DRC, Senegal, and Uganda, a smaller percentage of females had received at least one vaccine dose compared with males, but these differences are not very large. A larger percentage of females than males reported that they had received at least one dose in Nigeria.

**Figure 2:**
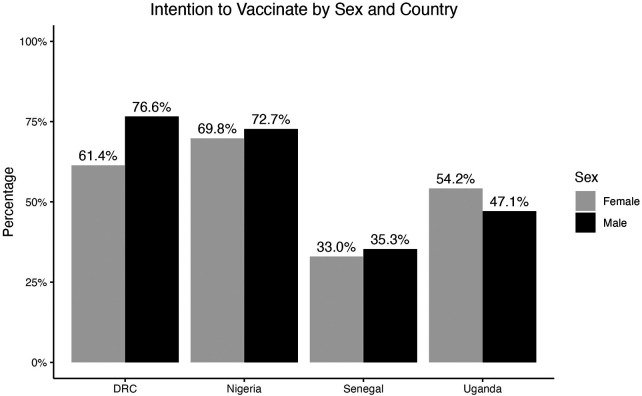
Percentage of respondents who reported that they intended to vaccinate, separated by country and sex.

Among respondents who were unvaccinated, 825 (63.4%) of males and 541 (54.0%) of females intended to be vaccinated against COVID-19. Figure 2 shows the percentages of males and females who reported that they intended to vaccinate by country. DRC and Nigeria had the highest percentages of respondents who intended to vaccinate, with 61.4% of females and 76.6% of males intending to vaccinate in the DRC, and 69.8% of females and 72.7% of males intending to vaccinate in Nigeria. Senegal had the lowest percentage of respondents who intended to vaccinate, with 33.0% of females and 35.3% of males reporting vaccination intention. In DRC, Nigeria, and Senegal, a higher percentage of males intended to vaccinate than females, and the difference was particularly high in the DRC.

**Figure 3:**
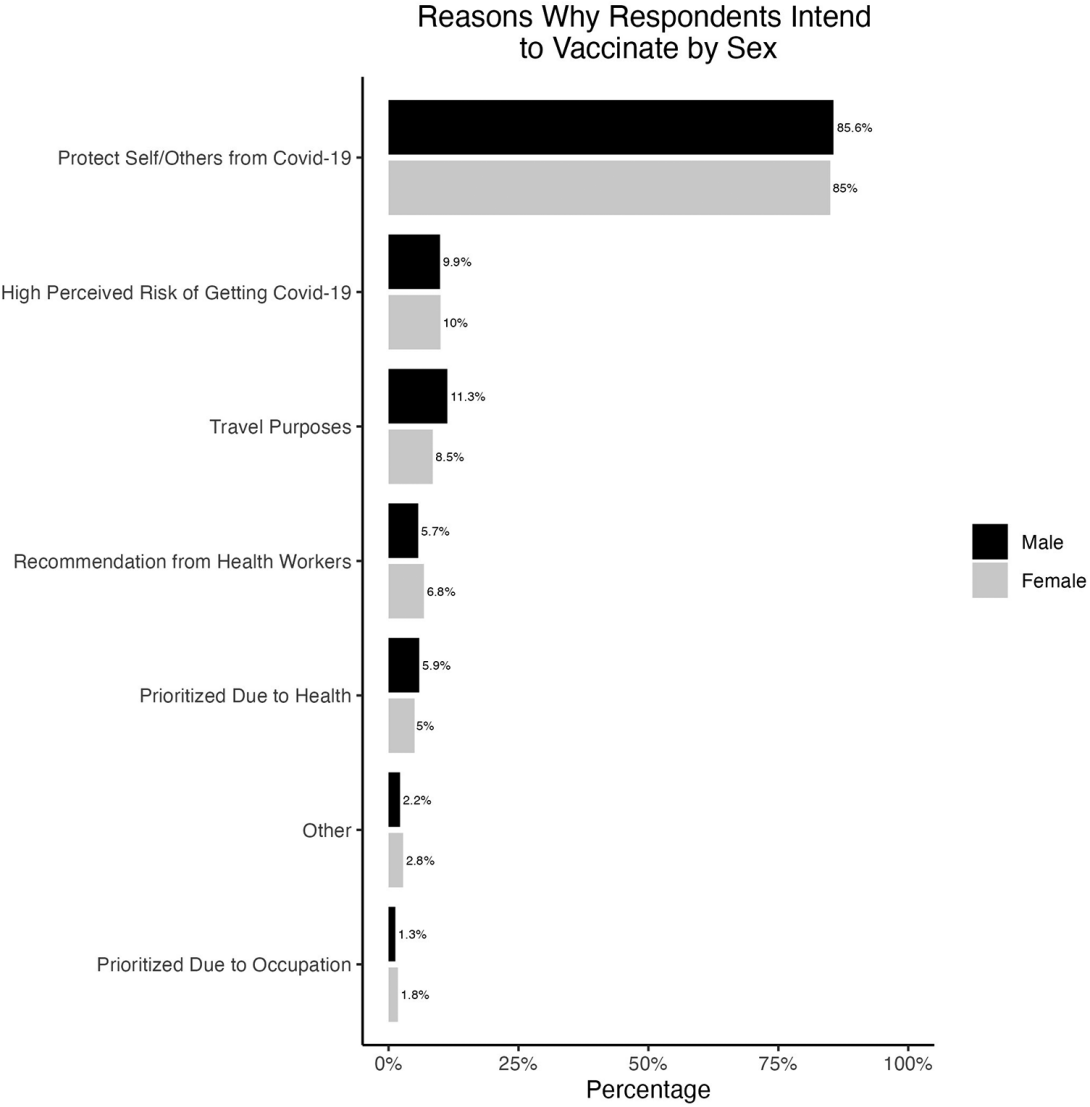
Reasons why respondents intended to vaccinate among those who had not yet vaccinated, separated by sex.

**Figure 4:**
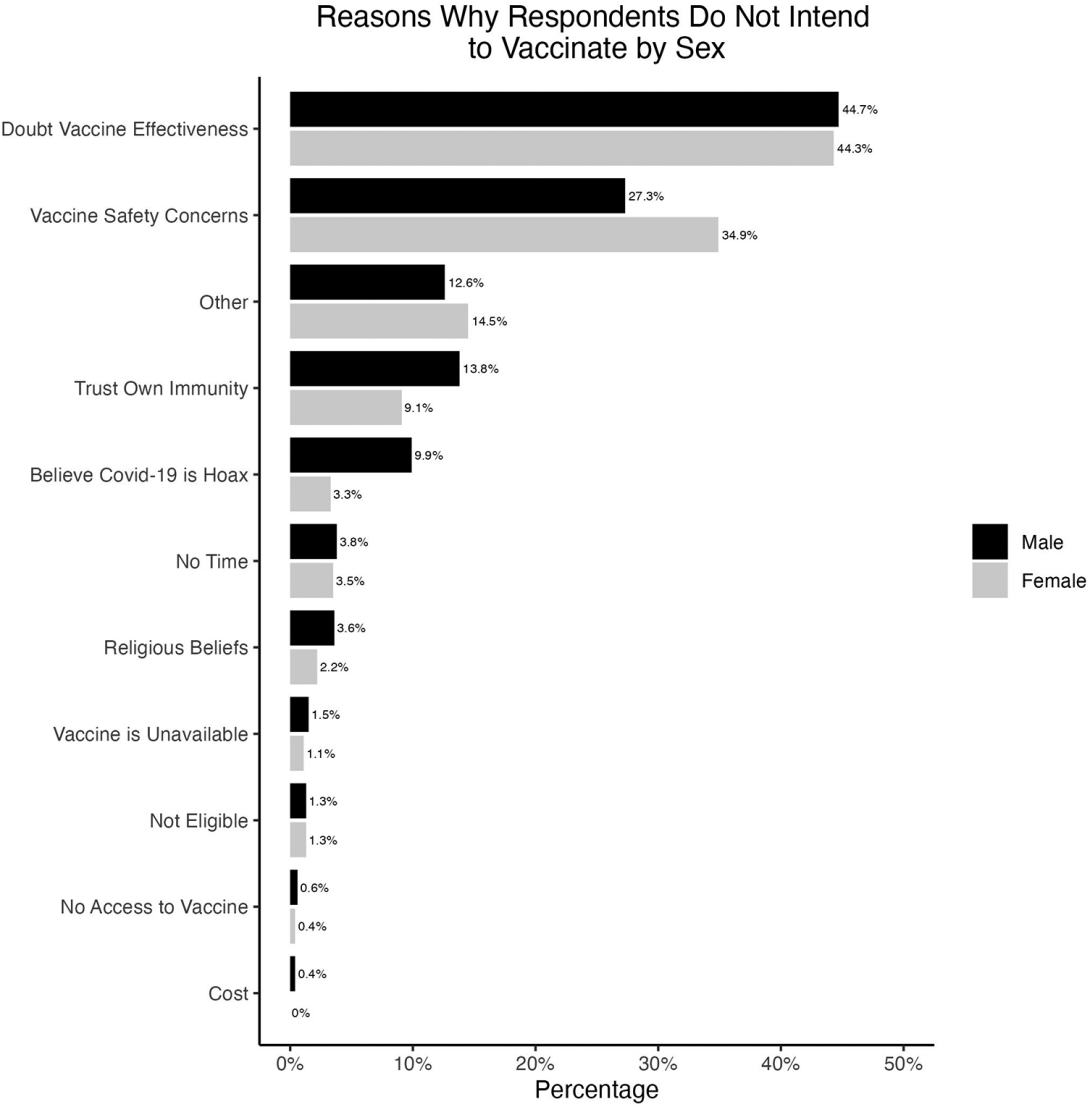
Reasons why respondents did not intend to vaccinate among those who were not vaccinated, separated by sex.

Respondents who intended to vaccinate and who did not intend to vaccinate were asked for reasons behind these intentions. The most common response for why they intended to vaccinate was to protect themselves from COVID-19 among both males and females (Figure 3). Some key reasons reported for the lack of intention to vaccinate was due to doubting vaccine effectiveness and safety among both males and females, and females were more concerned about vaccine safety (34.9%) than males (27.3%) (Figure 4).

### 3.3 Relationship between sex and COVID-19 vaccination uptake and intention

The relationship between sex and vaccination uptake yielded different results than the relationship between sex and vaccination intention. Table 2 reports the results for the correlations between sex and vaccination uptake and intention (see referenced Supplementary Tables S3, S5 for full results from each model). A multivariate analysis that controlled for age, residence, socio-economic index, education, and employment found that uptake of COVID-19 vaccines was not significantly different between males and females [AOR = 1.08 (95% CI: 0.87, 1.34), *p* = 0.47] (see Supplementary Table S5). Compared to males, females were slightly more likely to be vaccinated, though not enough to be significantly different from males overall.

In contrast, COVID-19 vaccination intention was significantly lower among females compared to males [AOR = 0.73 (95% CI: 0.58, 0.92), *p* = 0.008].

A stratified analysis comparing males to females in the 40 years and below category and those above 40 years showed that females aged 40 years and below had a significantly lower intention to vaccinate compared to males in the same age category [AOR = 0.69 (95% CI: 0.54, 0.89), *p* = 0.004] (Supplementary Table S4). However, there was no significant difference between males and females aged above 40 years (Supplementary Table S4).

### 3.4 COVID-19 vaccination uptake separated by sex

By studying males and females separately, results show that factors that promoted vaccination uptake were similar by sex. For both males and females, respondent's age, having heard of any COVID-19 vaccines, having someone who had been vaccinated in the household, and the belief that vaccines were distributed fairly were significantly associated with higher COVID-19 vaccination uptake. In contrast, for both males and females, being a current student or prior refusal to vaccinate were significantly associated with lower COVID-19 vaccination uptake. Among males, those who obtained COVID-19 information from health workers [AOR = 1.33 (95% CI: 1.02, 1.75), *p* = 0.04], those who had ever tested for COVID-19 [AOR = 2.62 (95% CI: 1.36, 5.06), *p* = 0.004] and those who trusted the Ministry of Health [AOR = 1.49 (95% CI: 1.01, 2.21), *p* = 0.045] were more likely to be vaccinated. For females, the odds of COVID-19 vaccination uptake were 1.45 times higher among those who had a high trust in the government index [AOR = 1.45 (95% CI: 1.04, 2.02), *p* = 0.03] (Table 3).

**Table 3:** Factors associated with COVID-19 vaccination uptake among males and females.

	Male			Female		
Variables	Vax *n* (%)	OR (95% CI)	AOR (95% CI)	Vax *n* (%)	OR (95% CI)	AOR (95% CI)
Age						
18–35	532 (44.3)	1	1	400 (36.9)	1	1
36–55	496 (48.6)	1.55 (1.25, 1.91)***	**1.47** (1.09, 1.97)**	385 (60.1)	2.37 (1.86, 3.02)***	**2.094** (1.467,2.99)***
56–65	116 (62.7)	2.72 (1.84, 4.01)***	**2.04** (1.14, 3.65)**	90 (65.2)	3.03 (1.95, 4.69)***	**1.909** **(1.001,3.64)**
66+	89 (65.4)	3.16 (2.05, 4.85)***	**4.30** (2.10, 8.82)***	53 (74.6)	4.01 (2.03, 7.89)***	**7.763** (2.382,25.294)**
Trust in academic institutions index						
Low	133 (29.7)	1	1	95 (27.6)	–	–
High	982 (52.4)	1.67 (1.28, 2.17)***	1.31 (0.90,1.90)	695 (52.3)	–	–
Education						
No School/illiterate	40 (43.0)	1	1	70 (57.9)	1	1
Other	38 (42.7)	1.28 (0.65, 2.52)	0.94 (0.27, 3.31)	22 (45.8)	0.97 (0.44, 2.12)	0.42 (0.08, 2.29)
Primary	246 (71.5)	1.56 (0.90, 2.72)	1.55 (0.52, 4.61)	195 (71.2)	0.97 (0.57, 1.65)	0.78 (0.28, 2.20)
Secondary	471 (45.6)	1.48 (0.90, 2.43)	1.70 (0.61, 4.70)	335 (40.7)	0.52 (0.33, 0.82)**	0.85 (0.33, 2.21)
Tertiary/Postgraduate	426 (44.2)	1.10 (0.67, 1.81)	1.39 (0.50, 3.86)	284 (44.4)	0.49 (0.31, 0.77)**	0.81 (0.31, 2.14)
Employment						
Some Employment	1,037 (50.3)	1	1	652 (56.3)	1	1
Student	43 (24.6)	0.43 (0.29,0.66)***	**0.44** (0.25, 0.76)**	44 (17.5)	0.25 (0.16, 0.37)***	**0.46** (0.26, 0.80)**
Unemployed/Retired/Housewife	140 (49.1)	1.27 (0.95, 1.69)	0.91 (0.60, 1.38)	217 (43.0)	0.72 (0.55, 0.93)**	0.83 (0.56, 1.21)
Declined vaccine						
No	1,132 (51.6)	1	1	846 (51.4)	1	1
Yes	101 (28.9)	0.35 (0.26, 0.48)***	**0.34** (0.23, 0.50)***	82 (28.5)	0.45 (0.33, 0.62)***	**0.531** (0.345,0.818)**
Heard of any COVID-19 vaccines						
No	14 (12.4)	1	1	11 (9.8)	1	1
Yes	1,219 (50.2)	5.39 (2.93, 9.91)***	**13.47** (4.34, 41.81)***	917 (50.3)	7.06 (3.63, 13.72)***	**26.86** (5.86, 123.17)***
Someone in the household was vaccinated						
No	221 (21.3)	1	1	182 (24.8)	1	1
Yes	990 (68.1)	4.42 (3.56, 5.50)***	**4.95** (3.70, 6.63)***	731 (62.4)	3.65 (2.83, 4.70)***	**3.80** (2.67, 5.40)***
Believe vaccines were fairly distributed						
Unfairly Distributed	168 (29.1)	1	1	113 (28.0)	1	1
Fairly Distributed	995 (61.0)	3.05 (2.38, 3.92)***	**2.21** (1.62, 3.02)***	734 (60.0)	3.00 (2.26,4.00)***	**2.10** (1.43, 3.09)***
Community as information source for COVID-19						
No	911 (44.6)	1	1	676 (43.6)	1	1
Yes	322 (64.8)	1.39 (1.06, 1.83)**	1.00 (0.68, 1.49)	252 (65.8)	1.48 (1.09, 2.00)**	1.06 (0.68, 1.64)
Family/friends as information source for COVID-19						
No	954 (48.3)	–	–	716 (48.7)	1	1
Yes	279 (49.5)	–	–	212 (45.5)	0.74 (0.58, 0.96)**	1.00 (0.70, 1.43)
Healthworkers as information source for COVID-19						
No	510 (40.2)	1	1	398 (40.9)	1	1
Yes	723 (56.8)	1.63 (1.34, 1.99)***	**1.33** (1.02, 1.75)*	530 (55.1)	1.39 (1.11,1.74)**	0.99 (0.71, 1.37)
Local leaders as information source for COVID-19						
No	934 (44.0)	1	1	702 (43.7)	1	1
Yes	299 (71.5)	1.72 (1.27, 2.32)***	1.38 (0.91, 2.07)	226 (69.1)	1.48 (1.042, 2.09)**	1.31 (0.82, 2.09)
Phone/social media/internet as information source for COVID-19						
No	638 (54.3)	1	1	530 (54.4)	1	1
Yes	595 (43.5)	0.71 (0.59, 0.87)**	0.86 (0.63, 1.16)	398 (41.4)	0.68 (0.55, 0.85)**	0.87 (0.62, 1.23)
Tested for COVID-19						
No	1,153 (47.7)	1	1	881 (47.4)	1	1
Yes	79 (65.3)	2.80 (1.78, 4.41)***	**2.62** (1.36, 5.06)**	43 (58.9)	1.86 (1.03, 3.36)*	1.65 (0.71, 3.82)
Trust scientific evidence						
Distrust	75 (34.6)	1	1	61 (34.1)	1	1
Trust	1,061 (51.0)	1.58 (1.11, 2.24)**	1.02 (0.64, 1.65)	746 (50.3)	1.70 (1.15, 2.51)**	1.15 (0.67, 1.97)
Trust in government index						
Low	620 (45.7)	–	–	415 (42.2)	1	1
High	597 (51.7)	–	–	490 (53.6)	1.60 (1.28, 2.01)***	**1.45** (1.04, 2.02)**
Trust WHO						
Distrust	95 (31.8)	1	1	58 (27.2)	1	1
Trust	1,031 (51.5)	1.84 (1.35, 2.51)***	1.13 (0.73, 1.75)	709 (50.5)	1.85 (1.27, 2.69)***	1.04 (0.60, 1.79)
Trust in ministry of health index						
Low	132 (28.6)	1	1	94 (25.1)	1	1
High	1,069 (53.6)	2.38 (1.83, 3.11)***	1.49 (1.01, 2.21)*	786 (53.8)	2.02 (1.50, 2.71)***	1.17 (0.75, 1.83)
Truthfulness of institutions index						
Low	119 (25.3)	1	1	86 (23.9)	1	1
High	1,102 (54.0)	2.13 (1.65, 2.75)***	1.38 (0.94, 2.04)	818 (53.7)	2.07 (1.54, 2.79)***	1.38 (0.87, 2.21)

Significant variables are highlighted for AOR only.

OR, odds ratio; AOR, adjusted odds ratio.

* *p* ≤ 0.05.

** *p* ≤ 0.01.

*** *p* < 0.001.

Bold indicates statistically significant in adjusted model.

### 3.5 COVID-19 vaccination intention separated by sex

Again, by studying males and females separately, the analysis shows whether the factors that correlate with intention to vaccinate among males are similar to the factors that correlate with intentions to vaccinate among females. Results showed only two factors were associated with vaccination intention among both males and females: having someone in the household who had been vaccinated against COVID-19 and trust in the Ministry of Health. Males who resided in semi-urban areas [AOR = 1.91 (95% CI: 1.13, 3.24) *p* = 0.02] had a significantly higher vaccination intention compared to their counterparts in urban areas while females who resided in rural areas [AOR = 2.02 (95% CI: 1.27, 3.20), *p* = 0.003] were the ones with significantly higher vaccination intention compared to those in urban areas. Among males, those who were from households with a higher socio-economic index [AOR = 0.64 (95%CI: 0.41, 0.99), *p* = 0.05] and those who had declined a vaccine before [AOR = 0.46 (95% CI: 0.32, 0.66), *p* = <0.001] had a significantly lower vaccination intention. On the other hand, males who had a higher trust in the WHO [AOR = 1.66 (95% CI: 1.11, 2.49), *p* = 0.01] and those who had a high truthfulness index [AOR = 1.71 (95% CI: 1.18, 2.46), *p* = 0.004] had a higher intention to vaccinate. Among females, there were no additional factors that were significantly associated with vaccination intention (Table 4).

**Table 4:** Factors associated with COVID-19 vaccination intention among males and females.

	Male			Female		
Variables	Will Vax *n* (%)	OR (95% CI)	AOR (95% CI)	Will Vax *n* (%)	OR (95% CI)	AOR (95% CI)
Age						
18–35	402 (60.5)	1	1	364 (53.5)	1	1
36–55	345 (66.1)	1.02 (0.79, 1.33)	0.99 (0.72, 1.37)	143 (55.9)	1.04 (0.77, 1.41)	0.97 (0.66, 1.43)
56–65	52 (76.5)	1.82 (0.98, 3.38)	1.62 (0.74, 3.51)	25 (52.1)	0.85 (0.46,1.58)	1.12 (0.49, 2.54)
66+	26 (55.3)	0.72 (0.38, 1.37)	0.61 (0.27, 1.41)	9 (50.0)	0.82 (0.31,2.18)	0.87 (0.22,3.45)
Residence						
Urban	425 (57.3)	1	1	304 (48.7)	1	1
Rural	287 (71.6)	1.26 (0.93,1.71)	1.43 (0.98, 2.10)	176 (68.5)	1.59 (1.13,2.25)**	**2.02** (1.27,3.20)**
Semi-urban	111 (72.1)	1.65 (1.09, 2.49)*	**1.91** (1.13, 3.24)**	61 (51.3)	1.09 (0.72,1.65)	1.02 (0.61,1.68)
Socio-economic index						
Low	220 (68.5)	1	1	136 (57.4)	–	–
Medium	327 (64.0)	0.81 (0.59, 1.12)	0.85 (0.56, 1.30)	232 (54.5)	–	–
High	276 (59.0)	0.57 (0.41, 0.80)**	**0.64** (0.41, 0.99)*	172 (51.5)	–	–
Education						
No school/illiterate	19 (35.8)	1	1	23 (45.1)	1	1
Other	28 (54.9)	1.32 (0.57, 3.05)	1.24 (0.36, 4.27)	10 (38.5)	0.56 (0.20, 1.53)	1.09 (0.22, 5.35)
Primary	60 (61.2)	1.49 (0.71, 3.11)	0.89 (0.29, 2.76)	46 (59.0)	1.24 (0.59, 2.63)	0.99 (0.25, 4.01)
Secondary	399 (71.1)	1.52 (0.80, 2.88)	1.14 (0.43, 2.99)	293 (60.0)	1.08 (0.58, 2.01)	1.26 (0.35, 4.50)
Tertiary/Postgraduate	317 (59.5)	1.07 (0.57, 2.01)	0.94 (0.36, 2.47)	167 (47.3)	0.71 (0.38, 1.33)	1.00 (0.28,3.61)
Declined vaccine						
No	714 (67.6)	1	1	446 (56.0)	–	–
Yes	111 (45.1)	0.46 (0.34, 0.62)***	**0.46** (0.32, 0.66)***	95 (46.6)	–	–
Heard of any COVID-19 vaccines						
No	61 (61.6)	–	–	41 (41.4)	1	1
Yes	764 (63.5)	–	–	500 (55.4)	1.60 (1.03, 2.50)*	1.14 (0.64,2.03)
Someone in the household was vaccinated						
No	536 (65.7)	1	1	297 (53.9)	1	1
Yes	272 (59.0)	1.60 (1.20, 2.15)**	**1.75** (1.23, 2.49)**	239 (54.6)	1.97 (1.44, 2.71)**	**1.93** (1.31, 2.86)**
Phone/social media/internet as information source for COVID-19						
No	362 (67.8)	1	1	249 (56.3)	–	–
Yes	463 (60.3)	0.77 (0.6,0.99)*	1.12 (0.80,1.56)	292 (52.1)	–	–
Trust scientific evidence						
Distrust	77 (54.2)	1	1	54 (45.8)	1	1
Trust	665 (65.6)	1.80 (1.23, 2.63)**	1.11 (0.71, 1.74)	413 (56.4)	1.58 (1.04, 2.38)*	1.03 (0.63, 1.70)
Trust in government index						
Low	448 (60.9)	1	1	303 (53.5)	–	–
High	373 (67.3)	1.61 (1.25, 2.08)***	1.01 (0.74, 1.39)	234 (55.3)	–	–
Trust WHO						
Distrust	75 (36.9)	1	1	59 (38.1)	1	1
Trust	676 (69.9)	2.97 (2.11, 4.17)***	**1.66** (1.12, 2.49)*	397 (57.6)	1.80 (1.24, 2.63)**	1.38 (0.87,2.19)
Trust in ministry of health index						
Low	159 (48.3)	1	1	120 (42.7)	1	1
High	638 (69.3)	2.58 (1.95, 3.41)***	**2.03** (1.43, 2.89)***	399 (59.4)	2.14 (1.58, 2.88)***	**1.70** (1.17, 2.48)**
Truthfulness of institutions index						
Low	166 (47.3)	1	1	117 (42.7)	1	1
High	651 (69.7)	2.76 (2.10, 3.62)***	**1.71** (1.18, 2.46)**	412 (58.8)	2.10 (1.55, 2.83)***	1.45 (0.97, 2.17)

Significant variables are highlighted for AOR only.

OR, odds ratio; AOR, adjusted odds ratio.

* *p* ≤ 0.05.

** *p* ≤ 0.01.

*** *p* < 0.001.

Bold indicates statistically significant in adjusted model.

## 4 Discussion

This study compared COVID-19 vaccine uptake and intention to vaccinate between males and females in the DRC, Nigeria, Senegal, and Uganda. This study found that while vaccination uptake was not significantly different between sexes, vaccination intention was significantly lower among females compared to males. The factors associated with vaccine uptake among males were obtaining COVID-19 information from health workers, having ever tested for COVID-19, and trust in the Ministry of Health while a high trust in the government predicted vaccine uptake among females. Having someone in the household who had been vaccinated against COVID-19 was associated with vaccine intention for both sexes. Among males, vaccination intention was associated with residing in a semi-urban area, a high socio-economic index, prior refusal of a vaccine, trust in the WHO, and a high truthfulness index. Among females, those who resided in rural areas had a higher vaccination intention compared to those in urban areas.

This study found that females had a significantly lower vaccination intention compared to males in line with previous reviews (7, 13, 14) including in Africa (5, 15, 16), and in cross-country analysis (17, 18). In Africa, studies in Cameroon (19), Ghana (20) and Zimbabwe (21) have also reported a lower vaccination intention among females. The difference in vaccine intention between males and females could be attributed to concerns related to vaccine safety (5, 15, 16, 18, 22), especially on the potential impact on reproduction (14, 22). Moreover, females have been found to report more concerns related to the vaccine side effects (23) and males have a more positive attitude towards COVID-19 vaccines (15).

On further analysis, it appears that the relationship between sex and vaccination intention was driven by age where younger females had a significantly lower vaccination intention when compared to younger males while there was no statistically significant difference among older females and males. It is possible that younger females could have had reproductive health concerns, such as hearing about myths associating vaccines with sub-fertility, that could have contributed to their vaccine hesitancy. Male counterparts did not have these safety concerns. Indeed, age remains an important predictor of vaccine intention across most studies in sub-Saharan Africa (5, 14, 17, 20). Other studies have found a higher vaccination acceptance among females who have had a high number of children (24), however, we were unable to examine this hypothesis in our study as we did not collect data on the number of children a woman had had. Moreover, in this study, whereas reasons for vaccine intention were similar among males and females, for lack of intention to vaccinate, younger females reported more vaccine safety concerns compared to the men in their age group. Addressing safety concerns could go a long way in improving vaccine intention among younger females.

Overall, the relationship between sex and COVID-19 vaccine intention requires a detailed examination to reduce potential for disparities in uptake of vaccines. For vaccine uptake, there was no significant difference between males and females in this study unlike in previous studies in Tanzania (25), Malawi (26), and Ghana (27) where uptake of vaccines was significantly among males. In our study countries, prioritization of COVID-19 vaccine access was for older age groups initially which could have accounted for similar uptake among both sexes, contributing to the observed result.

The key factors that influenced vaccine intention and uptake among males and females were related to trust and truthfulness similar to the literature (15, 21, 28). Indeed, among both males and females, trust in the Ministry of Health and beliefs that institutions were being truthful about COVID-19 were important factors. The most reported reason for vaccine hesitancy was doubting the vaccine effectiveness and safety concerns as reported in previous literature (15, 18, 23, 29). Misinformation or conflicting information about the vaccine from the media has also been implicated in vaccine hesitancy (15). Our research emphasizes the need to build trust in the vaccine and in the MOH, WHO, and other public health agencies. Providing factual, consistent, and effective communication through trusted channels and dealing with vaccine misinformation could go a long way in achieving more trust in government and international agencies.

Among males, those with a high socio-economic index household were less likely to intend to vaccinate. Also, males who reported obtaining COVID-19 information from health workers and those who had previously tested for COVID-19 had a higher vaccine uptake. Most previous studies have found a negative relationship with a high socio-economic index (14, 18, 29). The findings that respondents who reported relying on health workers as a source of COVID-19 information (30) and those who had tested for the virus (a proxy for perceived susceptibility or personal urgency) (28) were more likely to be vaccinated is consistent with the literature. This study highlights the potential to capitalize on trusted sources of information such as health workers to increase vaccine intention and uptake.

Males residing in a semi-urban area were more likely to intend to vaccinate. For females, the additional factor associated with vaccination intention was residence in rural areas. Residence in semi-urban areas for males and rural areas for females could be a proxy for vaccine reach as earlier vaccination efforts mostly targeted urban areas. Previous studies also reported a higher vaccine hesitancy among individuals residing in urban areas (18, 29). Therefore, extending vaccines to rural and semi-urban areas and addressing vaccine safety concerns through trusted guarantors would result in higher vaccine uptake among both males and females.

This study was conducted in four sub-Saharan African countries. However, as this was a phone survey, there was a high potential for selection bias as only those with access to phones could participate. The study samples were also constituted based on the COVID-19 infection statistics during the pandemic and thus males and urban residents had a higher representation. The reporting of vaccination intention and uptake were subject to social desirability bias which we minimized by seeking vaccination details regarding place of vaccination and vaccine received from respondents. We also did not collect data on comorbidity which variable would have been informative for our analysis. Overall, the study provides deeper analysis of the factors that could influence uptake of COVID-19 vaccines among males and females in four African countries, which can guide policy and practice.

## 5 Conclusions

The study found that less than half of males and females had been vaccinated against COVID-19. Among those unvaccinated, two-thirds of males and just over half of females intended to be vaccinated. Whereas uptake of COVID-19 vaccines was not different between males and females, intention to vaccinate was significantly higher among males. Overall, the key factors differentiating vaccine uptake and intention to vaccinate among males and females were mostly related to trust in government institutions, perceived truthfulness of institutions, and respondent's residence. To increase vaccine uptake among both males and females, there is a need to provide factual, consistent, and effective information about vaccines and address any safety concerns through trusted guarantors as well as extend vaccine access to rural and semi-urban areas.

## Data Availability

The raw data supporting the conclusions of this article will be made available by the authors, without undue reservation.

## References

[1] Watson OJ Barnsley G Toor J Hogan AB Winskill P Ghani AC. Global impact of the first year of COVID-19 vaccination: a mathematical modelling study. Lancet Infect Dis. (2022) 22(9):1293–302. 10.1016/S1473-3099(22)00320-6 35753318 PMC9225255

[2] Asundi A O’Leary C Bhadelia N. Global COVID-19 vaccine inequity: the scope, the impact, and the challenges. Cell Host Microbe. (2021) 29(7):1036–9. 10.1016/j.chom.2021.06.007 34265241 PMC8279498

[3] Loembé MM Nkengasong JN. COVID-19 vaccine access in Africa: global distribution, vaccine platforms, and challenges ahead. Immunity. (2021) 54(7):1353–62. 10.1016/j.immuni.2021.06.017 34260880 PMC8276532

[4] Moucheraud C Phiri K Whitehead HS Songo J Lungu E Chikuse E Uptake of the COVID-19 vaccine among healthcare workers in Malawi. Int Health. (2023) 15(1):77–84. 10.1093/inthealth/ihac007 35294960 PMC9808523

[5] Naidoo D Meyer-Weitz A Govender K. Factors influencing the intention and uptake of COVID-19 vaccines on the African continent: a scoping review. Vaccines (Basel). (2023) 11(4):873. 10.3390/vaccines11040873 37112785 PMC10146577

[6] Osuagwu UL Langsi R Ovenseri-Ogbomo G Mashige KP Abu EK Envuladu EA analysis of perception, reasons, and motivations for COVID-19 vaccination in people with diabetes across sub-saharan Africa: a mixed-method approach. Int J Environ Res Public Health. (2022) 19(13):7875. 10.3390/ijerph19137875 35805551 PMC9266073

[7] Wang Q Hu S Du F Zang S Xing Y Qu Z Mapping global acceptance and uptake of COVID-19 vaccination: a systematic review and meta-analysis. Commun Med (Lond). (2022) 2:113. 10.1038/s43856-022-00177-6 36101704 PMC9465145

[8] Heidari S Durrheim DN Faden R Kochhar S MacDonald N Olayinka F Time for action: towards an intersectional gender approach to COVID-19 vaccine development and deployment that leaves no one behind. BMJ Global Health. (2021) 6(8):e006854. 10.1136/bmjgh-2021-006854 34389628 PMC8366282

[9] Flor LS Friedman J Spencer CN Cagney J Arrieta A Herbert ME Quantifying the effects of the COVID-19 pandemic on gender equality on health, social, and economic indicators: a comprehensive review of data from march, 2020, to september, 2021. Lancet. (2022) 399(10344):2381–97. 10.1016/S0140-6736(22)00008-3 35247311 PMC8890763

[10] Wendt A Santos TM Cata-Preta BO Costa JC Mengistu T Hogan DR Children of more empowered women are less likely to be left without vaccination in low-and middle-income countries: a global analysis of 50 DHS surveys. J Glob Health. (2022) 12:1–9. 10.7189/jogh.12.04022 PMC8943525 35356658

[11] Ndejjo R Chen N Kabwama SN Namale A Wafula ST Wanyana I Uptake of COVID-19 vaccines and associated factors among adults in Uganda: a cross-sectional survey. BMJ open. (2023) 13(3):e067377. 10.1136/bmjopen-2022-067377 36931667 PMC10030279

[12] Beck N. Estimating grouped data models with a binary-dependent variable and fixed effects via a logit versus a linear probability model: the impact of dropped units. Polit Anal. (2020) 28(1):139–45. 10.1017/pan.2019.20

[13] Zintel S Flock C Arbogast AL Forster A von Wagner C Sieverding M. Gender differences in the intention to get vaccinated against COVID-19: a systematic review and meta-analysis. J Public Health. (2023) 31(8):1303–27. 10.1007/s10389-021-01677-w PMC8739532 35018277

[14] Robinson E Jones A Daly M. International estimates of intended uptake and refusal of COVID-19 vaccines: a rapid systematic review and meta-analysis of large nationally representative samples. Vaccine. (2021) 39(15):2024–34. 10.1016/j.vaccine.2021.02.005 33722411 PMC7867398

[15] Ackah BBB Woo M Stallwood L Fazal ZA Okpani A Ukah UV COVID-19 vaccine hesitancy in Africa: a scoping review. Global Health Research and Policy. (2022) 7(1):21. 10.1186/s41256-022-00255-1 35850783 PMC9294808

[16] Gudayu TW Mengistie HT. COVID-19 vaccine acceptance in sub-saharan African countries: a systematic review and meta-analysis. Heliyon. (2023) 9(2):e13037. 10.1016/j.heliyon.2023.e13037 36686610 PMC9846884

[17] Bono SA Faria de Moura Villela E Siau CS Chen WS Pengpid S Hasan MT Factors affecting COVID-19 vaccine acceptance: an international survey among low-and middle-income countries. Vaccines (Basel). (2021) 9(5):515. 10.3390/vaccines9050515 34067682 PMC8157062

[18] Wollburg P Markhof Y Kanyanda S Zezza A. The evolution of COVID-19 vaccine hesitancy in sub-saharan Africa: evidence from panel survey data. BMC Proc. (2023) 17(Suppl 7):8. 10.1186/s12919-023-00266-x 37415169 PMC10324117

[19] Amani A Mossus T Lekeumo Cheuyem FZ Bilounga C Mikamb P Basseguin Atchou J Gender and COVID-19 vaccine disparities in Cameroon. COVID. (2022) 2(12):1715–30. 10.3390/covid2120123

[20] Acheampong T Akorsikumah EA Osae-Kwapong J Khalid M Appiah A Amuasi JH. Examining vaccine hesitancy in sub-saharan Africa: a survey of the knowledge and attitudes among adults to receive COVID-19 vaccines in Ghana. Vaccines (Basel). (2021) 9(8):814. 10.3390/vaccines9080814 34451939 PMC8402404

[21] McAbee L Tapera O Kanyangarara M. Factors associated with COVID-19 vaccine intentions in eastern Zimbabwe: a cross-sectional study. Vaccines (Basel). (2021) 9(10):1109. 10.3390/vaccines9101109 34696215 PMC8538260

[22] Skjefte M Ngirbabul M Akeju O Escudero D Hernandez-Diaz S Wyszynski DF COVID-19 vaccine acceptance among pregnant women and mothers of young children: results of a survey in 16 countries. Eur J Epidemiol. (2021) 36(2):197–211. 10.1007/s10654-021-00728-6 33649879 PMC7920402

[23] Neumann-Böhme S Varghese NE Sabat I Barros PP Brouwer W van Exel J Once we have it, will we use it? A European survey on willingness to be vaccinated against COVID-19. Eur J Health Econ. (2020) 21:977–82. 10.1007/s10198-020-01208-6 32591957 PMC7317261

[24] Mekuriaw BY Nigatu D Dessie AM Asresie MB. Intention to take COVID-19 vaccine and associated factors among pregnant women attending antenatal care at public health facilities in Bahir Dar city, Northwest Ethiopia. BMC Women’s Health. (2023) 23(1):175. 10.1186/s12905-023-02331-1 37041619 PMC10088773

[25] Msuya SE Manongi RN Jonas N Mtei M Amour C Mgongo MB COVID-19 vaccine uptake and associated factors in sub-saharan Africa: evidence from a community-based survey in Tanzania. Vaccines (Basel). (2023) 11(2):465. 10.3390/vaccines11020465 36851342 PMC9961769

[26] Whitehead HS Songo J Phiri K Kalande P Lungu E Phiri S Correlates of uptake of COVID-19 vaccines and motivation to vaccinate among Malawian adults. Hum Vaccin Immunother. (2023) 19(2):2228168. 10.1080/21645515.2023.2228168 37394430 PMC10332229

[27] Nasiratu I Pencille LB Khuzwayo N Aboagye RG Tarkang EE. Predictors of COVID-19 vaccine uptake among persons aged 18 years and above in Ga North municipality, Ghana using the health belief model: a community-based cross-sectional study. PLoS One. (2023) 18(11):e0293350. 10.1371/journal.pone.0293350 37934776 PMC10629641

[28] Davis TP Yimam AK Kalam MA Tolossa AD Kanwagi R Bauler S Behavioural determinants of COVID-19-vaccine acceptance in rural areas of six lower- and middle-income countries. Vaccines (Basel). (2022) 10(2):214. 10.3390/vaccines10020214 35214672 PMC8875839

[29] Kanyanda S Markhof Y Wollburg P Zezza A. Acceptance of COVID-19 vaccines in sub-saharan Africa: evidence from six national phone surveys. BMJ Open. (2021) 11(12):e055159. 10.1136/bmjopen-2021-055159 34911723 PMC8678558

[30] Wollburg P Markhof Y Kanyanda S Zezza A. Assessing COVID-19 vaccine hesitancy and barriers to uptake in sub-saharan Africa. Commun Med. (2023) 3(1):121. 10.1038/s43856-023-00330-9 37696937 PMC10495410

