# Supplementary material for: Sex and COVID-19 vaccination uptake and intention in the Democratic Republic of Congo, Nigeria, Senegal, and Uganda
Source: Front Glob Womens Health. 2024 Jun 12;5:1356609. doi: 10.3389/fgwh.2024.1356609 (PMC11210425; doi:10.3389/fgwh.2024.1356609)
Supplement: Supplementary file 1 [file Datasheet1.docx]

# Supplementary Material

Supplementary Figure 1: Reasons why respondents vaccinated by sex


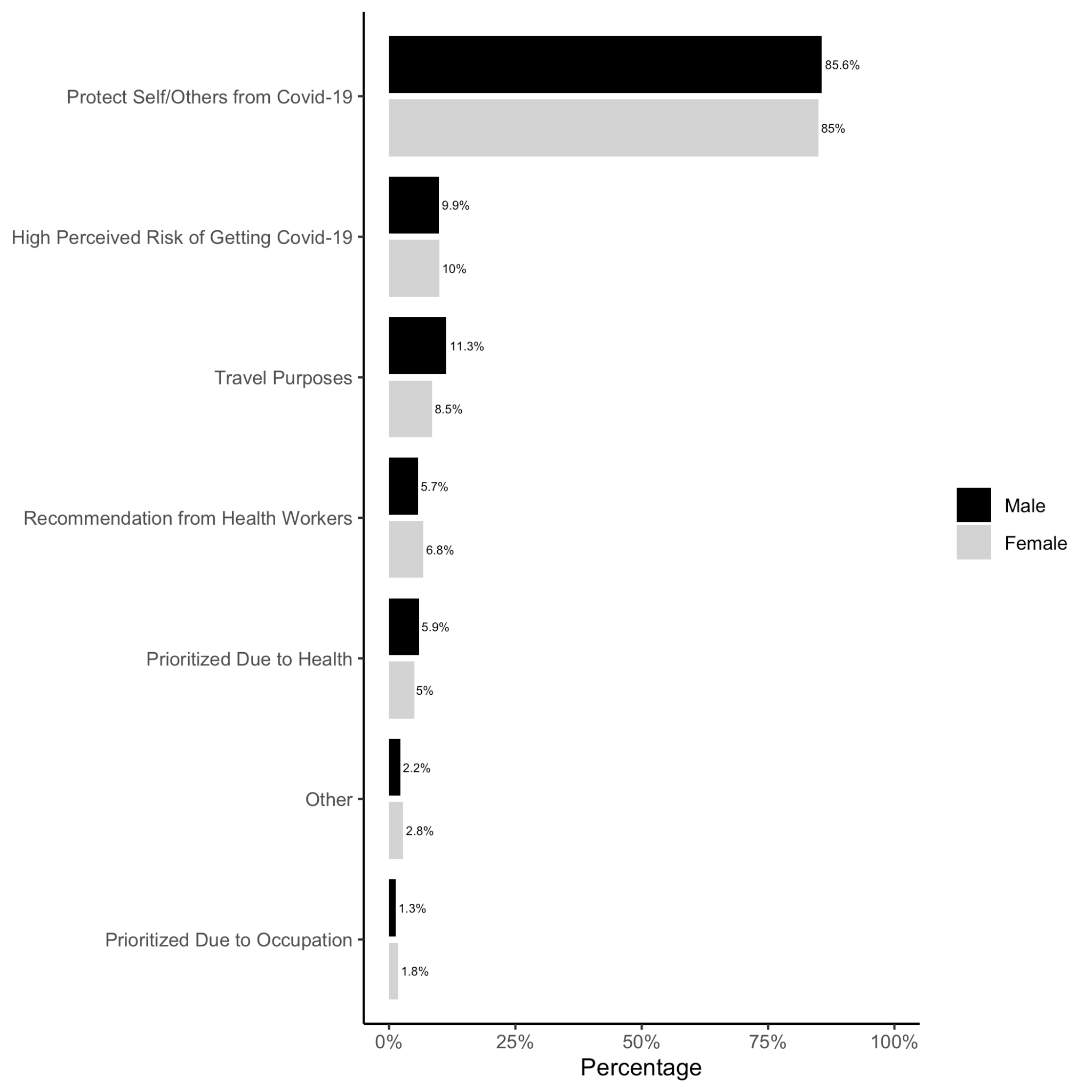


Supplementary Figure 2: Reasons why respondents did not get vaccinated by sex


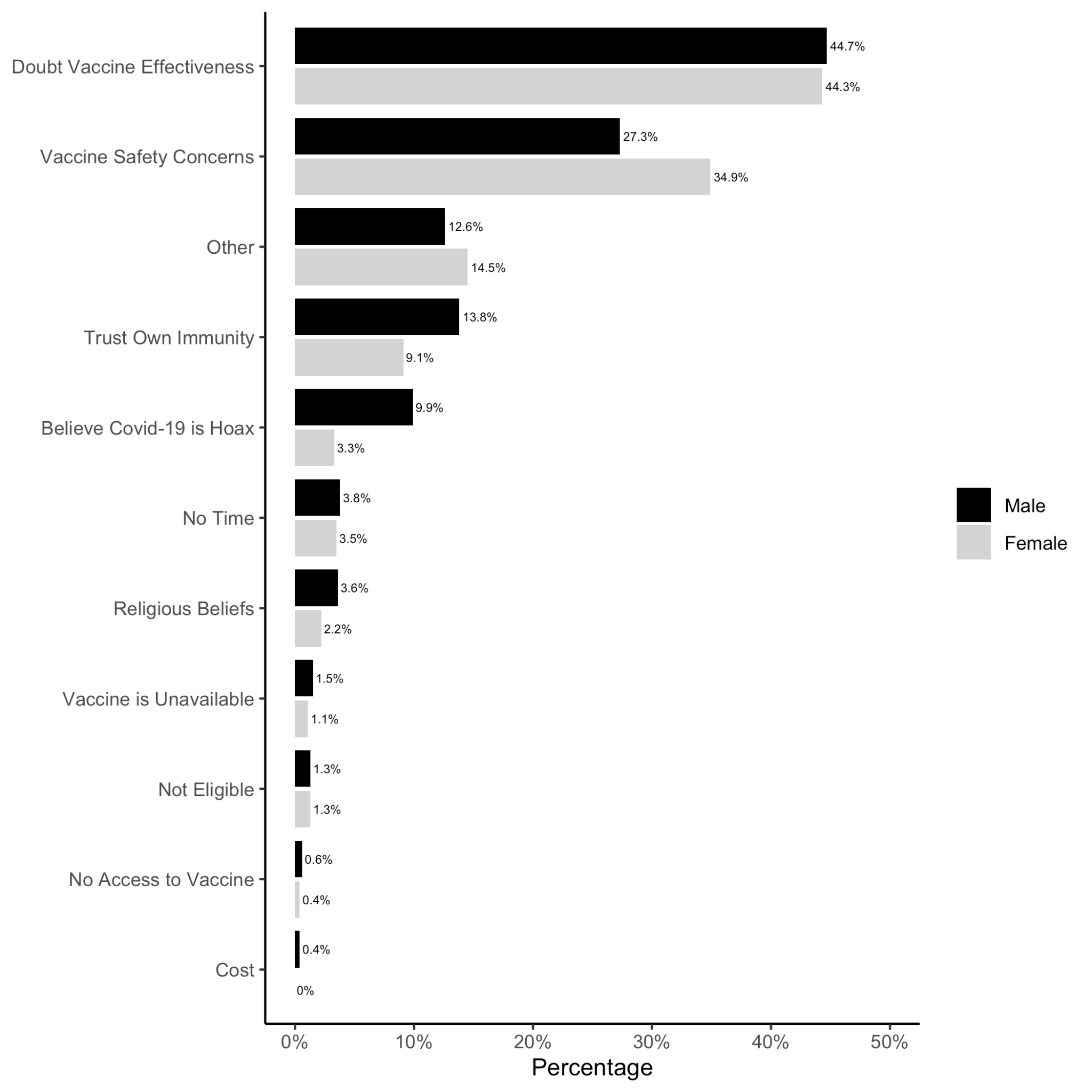


**Supplementary Table 1**

**Distributions for Quota Sampling by Country**

| **Country** | **Province** | **Sex** | **Age** | | | | | **Subtotal** |
| --- | --- | --- | --- | --- | --- | --- | --- | --- |
|  |  |  | **18-35 (51%)** | | **36-55 (37%)** | **56-65 (8%)** | **>65 (4%)** |  |
| **Democratic Republic of the Congo  (n = 1058)** | Equateur  (n = 12) | Female | 2 | | 2 | 0 | 0 | 4 |
|  |  | Male | 4 | | 3 | 1 | 0 | 8 |
|  | Kasai  (n = 13) | Female | 3 | | 2 | 0 | 0 | 5 |
|  |  | Male | 4 | | 3 | 1 | 0 | 8 |
|  | Katanga  (n = 131) | Female | 24 | | 18 | 4 | 2 | 48 |
|  |  | Male | 42 | | 31 | 7 | 3 | 83 |
|  | Kivu  (n = 167) | Female | 32 | | 23 | 5 | 2 | 62 |
|  |  | Male | 54 | | 39 | 8 | 4 | 105 |
|  | Leopoldville  (n = 683) | Female | 129 | | 94 | 20 | 10 | 253 |
|  |  | Male | 219 | | 159 | 35 | 17 | 430 |
|  | Orientale  (n = 52) | Female | 10 | | 7 | 2 | 1 | 20 |
|  |  | Male | 16 | | 12 | 3 | 1 | 32 |
|  | **State** | **Sex** | **Age** | | | | | **Subtotal** |
|  |  |  | **18-24 (15%)** | **25-34 (27%)** | **35-49 (33%)** | **50-64 (17%)** | **≥65 (8%)** |  |
| **Nigeria  (n = 1048)** | Kaduna  (n = 95) | Female | 7 | 12 | 15 | 8 | 4 | 46 |
|  |  | Male | 9 | 12 | 16 | 8 | 4 | 49 |
|  | FCT  (n = 209) | Female | 17 | 22 | 17 | 12 | 8 | 76 |
|  |  | Male | 13 | 31 | 40 | 21 | 8 | 113 |
|  | Imo  (n = 56) | Female | 4 | 7 | 7 | 3 | 2 | 23 |
|  |  | Male | 5 | 8 | 12 | 6 | 2 | 33 |
|  | Lagos  (n = 499) | Female | 36 | 58 | 59 | 34 | 13 | 200 |
|  |  | Male | 37 | 75 | 110 | 54 | 23 | 299 |
|  | Gombe  (n = 43) | Female | 3 | 4 | 5 | 3 | 2 | 17 |
|  |  | Male | 3 | 7 | 9 | 5 | 2 | 26 |
|  | Rivers  (n = 146) | Female | 12 | 16 | 16 | 9 | 5 | 58 |
|  |  | Male | 12 | 23 | 32 | 16 | 5 | 88 |
|  | **Zone** | **Sex** | **Age** | | | | | **Subtotal** |
|  |  |  | **18-35 (55%)** | | **36-55 (31%)** | **56-65 (8%)** | **>65 (6%)** |  |
| **Senegal  (n = 1056)** | West (n = 804) | Female | 221 | | 124 | 32 | 24 | 401 |
|  |  | Male | 222 | | 124 | 33 | 24 | 403 |
|  | North (n = 77) | Female | 21 | | 13 | 4 | 1 | 39 |
|  |  | Male | 21 | | 12 | 4 | 1 | 38 |
|  | East (n = 18) | Female | 5 | | 3 | 1 | 0 | 9 |
|  |  | Male | 5 | | 3 | 1 | 0 | 9 |
|  | Centre (n = 110) | Female | 30 | | 17 | 5 | 4 | 56 |
|  |  | Male | 30 | | 17 | 5 | 2 | 54 |
|  | South (n = 47) | Female | 14 | | 7 | 2 | 1 | 24 |
|  |  | Male | 13 | | 7 | 2 | 1 | 23 |
|  | **Regions** | **Sex** | **Age** | | | | | **Subtotal** |
|  |  |  | **18-35 (51%)** | | **36-55 (37%)** | **56-65 (8%)** | **>65 (4%)** |  |
| **Uganda  (n = 1069)** | Northern  (n = 162) | Female | 33 | | 24 | 5 | 3 | 65 |
|  |  | Male | 49 | | 36 | 8 | 4 | 97 |
|  | Eastern  (n = 162) | Female | 33 | | 24 | 5 | 3 | 65 |
|  |  | Male | 49 | | 36 | 8 | 4 | 97 |
|  | Central  (n = 583) | Female | 118 | | 86 | 19 | 10 | 233 |
|  |  | Male | 178 | | 130 | 28 | 14 | 350 |
|  | Western  (n = 162) | Female | 33 | | 24 | 5 | 3 | 65 |
|  |  | Male | 49 | | 36 | 8 | 4 | 97 |

**Supplementary Table 2**

**Indices and Dependent Variable Measures**

| **Measure Type/Index Creation** | **Question** | **Response** |
| --- | --- | --- |
| **Vaccination Rate**  Dependent Variable | Have you received the COVID-19 vaccine? | Yes, full dose; Yes, incomplete dose; No; Refused |
| **Vaccination Intention**  Dependent Variable | Do you intend to receive the COVID-19 vaccine if it is available? | Yes, No, Refused |
| **Socioeconomic Index**  Index: Consolidate these 6 questions into an index by combining “Does your household have XXX” questions to create a measure that is the count out of 6 items. “Refuse” and “Don’t know” are considered NA.  “Low” – index is between 1-2  “Middle” – index is between 3-4  “Higher – index is between 5-6 | - Does your household have **a television**? - Does your household have **electricity**? - Does your household have **a computer**? - Does your household have a **sofa set**? - Does your household have a **refrigerator**? - Does anyone in your household own a **cassette/CD/DVD player**? | Yes;  No;  Don’t know;  Refused |
| **Trust in Academic Institutions Index**  Index: Consolidate these 7 statements into an index by reordering responses such that higher numbers indicate more positive attitudes toward Ministry of Health and combining by taking the mean of the responses. Index can range from 1-5. “Refuse” and “Don’t know” are considered NA.  “Low” – Index is between 1-3  “High” – Index is between 4-5 | **To what extent do you agree or disagree with the following statements?**   - Academic institutions are **competent (has knowledge and expertise)** in the COVID-19 response. - Academic institutions are **objective (no hidden agenda / good intentions)** in the COVID-19 response. - Academic institutions are **fair (all opinions were heard & considered)** in the COVID-19 response. - Academic institutions are **consistent (messages are aligned to action)** in the COVID-19 response. - Academic institutions are **sincere (transparent)** in the COVID-19 response. - Academic institutions are **faithful (empathetic, listens, and understands)** in the COVID-19 response. - Academic institutions are **well-resourced (has enough money/equipment)** in the COVID-19 response. | Strongly disagree;  Partially disagree;  Neither agree nor disagree;  Partially agree;  Strongly agree;  Don’t know  Refused |
| **Trust in Government Index**  Index: Consolidate these 4 statements into an index by reordering responses such that higher numbers indicate more belief in government competence and combining by taking the mean of the responses. “Refuse” and “Don’t know” are considered NA.  “Low” – Index is between 1-3  “High” – Index is between 4-5 | - Corruption is widespread in government. - Government authorities try their best to help citizens. - When government authorities really want to achieve something, they have enough resources and ability to do it. - When government authorities really want to achieve something, they usually accomplish it successfully. | Strongly disagree;  Partially disagree;  Neither agree nor disagree;  Partially agree;  Strongly agree;  Don’t know;  Refused |
| **Trust in Ministry of Health Index**  Index: Consolidate these 7 statements into an index by reordering responses such that higher numbers indicate more positive attitudes toward academic institutions and combining by taking the mean of the responses. “Refuse” and “Don’t know” are considered NA.  “Low” – Index is between 1-3  “High” – Index is between 4-5 | **To what extent do you agree or disagree with the following statements?**   - Ministry of Health is **competent (has knowledge and expertise)** in the COVID-19 response. - Ministry of Health is **objective (no hidden agenda / good intentions)** in the COVID-19 response. - Ministry of Health is **fair (all opinions were heard & considered)** in the COVID-19 response. - Ministry of Health is **consistent (messages are aligned to action)** in the COVID-19 response. - Ministry of Health is **sincere (transparent)** in the COVID-19 response. - Ministry of Health is **faithful (empathetic, listens, and understands)** in the COVID-19 response. - Ministry of Health is **well-resourced (has enough money/equipment)** in the COVID-19 response. | Strongly disagree;  Partially disagree;  Neither agree nor disagree;  Partially agree;  Strongly agree;  Don’t know  Refused |
| **Truthfulness of Institutions Index**  Index: Consolidate these 4 statements into an index by reordering responses such that higher numbers indicate more belief in truthfulness of the institution combining by taking the mean of the responses. “Refuse” and “Don’t know” are considered NA.  “Low” – Index is between 1-3  “High” – Index is between 4-5 | - How truthful or untruthful do you think **the government** have been about the COVID-19 outbreak in Uganda? - How truthful or untruthful do you think **scientists** have been about the COVID-19 outbreak in Uganda? - How truthful or untruthful do you think **traditional leaders** have been about the COVID-19 outbreak in Uganda? - How truthful or untruthful do you think **health workers** have been about the COVID-19 outbreak in Uganda? - How truthful or untruthful do you think **the Ministry of Health** have been about the COVID-19 outbreak in Uganda? - How truthful or untruthful do you think **the World Health Organization** have been about the COVID-19 outbreak in Uganda? | Very untruthful;  Somewhat untruthful;  Neither;  Somewhat truthful;  Very truthful;  Don’t know;  Refused |

**Supplementary Table 3**

**Association between sex and COVID-19 vaccination intent**

| **Variables** | **Will  Vax** | **Will  Not Vax** | **OR  (95% CI)** | **OR  *p*-value** | **Adjusted OR  (95% CI)** | **Adjusted OR  *p*-value** |
| --- | --- | --- | --- | --- | --- | --- |
| **Sex** | | | | | | |
| Male | 825 | 477 | -- | -- | -- | -- |
| Female | 541 | 461 | 0.714 (0.598,0.853) | **<0.001** | **0.729 (0.578,0.92)** | **0.008** |
|  |  |  |  |  |  |  |
| **Age** | | | | | | |
| 18-35 | 766 | 579 | -- | -- | -- | -- |
| 36-55 | 488 | 290 | 1.125 (0.928,1.364) | 0.231 | 1.001 (0.779,1.287) | 0.993 |
| 56-65 | 77 | 39 | 1.347 (0.883,2.055) | 0.166 | 1.526 (0.851,2.737) | 0.156 |
| 66+ | 35 | 30 | 0.802 (0.473,1.361) | 0.413 | 0.628 (0.308,1.279) | 0.2 |
|  |  |  |  |  |  |  |
| **Residence** | | | | | | |
| Urban | 729 | 637 | -- | -- | -- | -- |
| Rural | 463 | 195 | 1.432 (1.142,1.794) | 0.002 | **1.68 (1.251,2.256)** | **0.001** |
| Semi-urban | 172 | 101 | 1.375 (1.033,1.83) | 0.029 | 1.363 (0.948,1.958) | 0.094 |
|  |  |  |  |  |  |  |
| **Trust in Academic Institutions Index** | | | | | | |
| Low | 317 | 246 | -- | -- | -- | -- |
| High | 947 | 571 | 1.385 (1.122,1.71) | 0.002 | 0.854 (0.649,1.122) | 0.257 |
|  |  |  |  |  |  |  |
| **Assets Index** | | | | | | |
| Low | 356 | 202 | -- | -- | -- | -- |
| Medium | 559 | 378 | 0.869 (0.69,1.095) | 0.234 | 0.823 (0.603,1.122) | 0.218 |
| High | 448 | 354 | 0.674 (0.531,0.855) | 0.001 | **0.721 (0.521,0.997)** | **0.048** |
|  |  |  |  |  |  |  |
| **Education** | | | | | | |
| No School | 42 | 62 | -- | -- | -- | -- |
| Other | 38 | 39 | 0.981 (0.523,1.841) | 0.953 | 1.165 (0.432,3.146) | 0.763 |
| Primary | 106 | 70 | 1.351 (0.799,2.285) | 0.262 | 1.067 (0.423,2.692) | 0.891 |
| Secondary | 692 | 357 | 1.291 (0.828,2.012) | 0.259 | 1.254 (0.567,2.776) | 0.576 |
| Tertiary/Postgraduate | 484 | 402 | 0.912 (0.586,1.419) | 0.683 | 1.015 (0.458,2.254) | 0.97 |
|  |  |  |  |  |  |  |
| **Employment** | | | | | | |
| Some Employment | 975 | 551 | -- | -- | -- | -- |
| Student | 150 | 185 | 1.044 (0.788,1.385) | 0.763 | 1.081 (0.748,1.562) | 0.679 |
| Unemployed/Retired/Housewife | 236 | 195 | 0.78 (0.62,0.981) | 0.034 | 0.85 (0.627,1.151) | 0.293 |
|  |  |  |  |  |  |  |
| **Declined Vaccine** | | | | | | |
| No | 1160 | 693 | -- | -- | -- | -- |
| Yes | 206 | 244 | 0.562 (0.45,0.7) | <0.001 | **0.612 (0.467,0.802)** | **<0.001** |
|  |  |  |  |  |  |  |
| **Someone in the Household was Vaccinated** | | | | | | |
| No | 833 | 534 | -- | -- | -- | -- |
| Yes | 511 | 388 | 1.718 (1.388,2.126) | <0.001 | **1.821 (1.397,2.374)** | **<0.001** |
|  |  |  |  |  |  |  |
| **Healthworkers as Information Source for Covid-19** | | | | | | |
| No | 753 | 573 | -- | -- | -- | -- |
| Yes | 613 | 365 | 1.259 (1.05,1.511) | 0.013 | 0.991 (0.791,1.241) | 0.934 |
|  |  |  |  |  |  |  |
| **Trust Scientific Evidence** | | | | | | |
| Distrust | 131 | 129 | -- | -- | -- | -- |
| Trust | 1078 | 668 | 1.68 (1.273,2.217) | <0.001 | 1.099 (0.783,1.544) | 0.585 |
|  |  |  |  |  |  |  |
| **Trust in Government Index** | | | | | | |
| Low | 751 | 551 | -- | -- | -- | -- |
| High | 607 | 370 | 1.466 (1.219,1.762) | <0.001 | 0.979 (0.772,1.242) | 0.86 |
|  |  |  |  |  |  |  |
| **Trust WHO** | | | | | | |
| Distrust | 134 | 224 | -- | -- | -- | -- |
| Trust | 1073 | 583 | 2.361 (1.836,3.037) | <0.001 | **1.49 (1.096,2.025)** | **0.011** |
|  |  |  |  |  |  |  |
| **Trust in Ministry of Health Index** | | | | | | |
| Low | 279 | 331 | -- | -- | -- | -- |
| High | 1037 | 555 | 2.413 (1.969,2.956) | <0.001 | **1.9 (1.451,2.487)** | **<0.001** |
|  |  |  |  |  |  |  |
| **Truthfulness of Institutions Index** | | | | | | |
| Low | 283 | 342 | -- | -- | -- | -- |
| High | 1063 | 572 | 2.453 (2.007,2.999) | <0.001 | **1.649 (1.254,2.169)** | **<0.001** |
|  |  |  |  |  |  |  |

**Supplementary Table 4**

**Association between sex and COVID-19 vaccination intent stratified by age**

|  | **40 and under** | | | | | **Over 40** | | | | |
| --- | --- | --- | --- | --- | --- | --- | --- | --- | --- | --- |
| **Variables** | **Will Vax  40 and under** | **OR  (95% CI)** | **OR  *p*-value** | **AOR  (95% CI)** | **AOR  *p*-value** | **Will Vax  over 40** | **OR  (95% CI)** | **OR  *p*-value** | **AOR  (95% CI)** | **AOR *p*-value** |
| **Sex** |  |  |  |  |  |  |  |  |  |  |
| Male | 550 | -- | -- | -- | -- | 275 | -- | -- | -- | -- |
| Female | 420 | 0.735 (0.599,0.903) | 0.003 | **0.69 (0.537, 0.886)** | **0.004** | 121 | 0.686 (0.474,0.995) | 0.047 | 0.985 (0.553, 1.753) | 0.959 |
| **Residence** |  |  |  |  |  |  |  |  |  |  |
| Urban | 556 | -- | -- | -- | -- | 173 | -- | -- | -- | -- |
| Rural | 300 | 1.354 (1.035,1.773) | 0.027 | **1.693 (1.197, 2.394)** | **0.003** | 163 | 1.594 (1.044,2.435) | 0.031 | 1.529 (0.798, 2.93) | 0.2 |
| Semi-urban | 112 | 1.209 (0.867,1.688) | 0.263 | 1.277 (0.84, 1.942) | 0.252 | 60 | 1.92 (1.079,3.415) | 0.026 | 2.232 (0.904, 5.51) | 0.082 |
| **Academic Index** |  |  |  |  |  |  |  |  |  |  |
| High | 672 | 1.365 (1.071,1.74) | 0.012 | 0.868 (0.637, 1.183) | 0.37 | 275 | -- | -- | -- | -- |
| Low | 226 | -- | -- | -- | -- | 91 | -- | -- | -- | -- |
| **Assets Index** |  |  |  |  |  |  |  |  |  |  |
| High | 329 | 0.679 (0.515,0.896) | 0.006 | 0.74 (0.513, 1.068) | 0.108 | 119 | -- | -- | -- | -- |
| Low | 242 | -- | -- | -- | -- | 114 | -- | -- | -- | -- |
| Medium | 397 | 0.897 (0.684,1.176) | 0.43 | 0.873 (0.612, 1.246) | 0.455 | 162 | -- | -- | -- | -- |
| **Education** |  |  |  |  |  |  |  |  |  |  |
| No School | 23 | -- | -- | -- | -- | 19 | -- | -- | -- | -- |
| Other | 27 | 0.928 (0.413,2.084) | 0.856 | 1.69 (0.462, 6.18) | 0.428 | 11 | 0.874 (0.301,2.534) | 0.804 | 0.394 (0.052, 2.982) | 0.367 |
| Primary | 53 | 0.959 (0.473,1.941) | 0.906 | 0.945 (0.263, 3.395) | 0.931 | 53 | 2.005 (0.867,4.637) | 0.104 | 1 (0.22, 4.547) | 1 |
| Secondary | 503 | 1.107 (0.608,2.014) | 0.74 | 1.334 (0.452, 3.934) | 0.602 | 189 | 1.387 (0.671,2.869) | 0.378 | 0.65 (0.174, 2.424) | 0.521 |
| Tertiary/Postgraduate | 362 | 0.79 (0.434,1.436) | 0.439 | 1.144 (0.387, 3.377) | 0.808 | 122 | 0.916 (0.441,1.906) | 0.815 | 0.407 (0.106, 1.554) | 0.188 |
| **Employment** |  |  |  |  |  |  |  |  |  |  |
| Some Employment | 635 | -- | -- | -- | -- | 340 | -- | -- | -- | -- |
| Student | 146 | -- | -- | -- | -- | 4 | 0.423 (0.125,1.432) | 0.167 | 0.756 (0.16, 3.561) | 0.723 |
| Unemployed/Retired/Housewife | 187 | -- | -- | -- | -- | 49 | 0.537 (0.337,0.854) | 0.009 | **0.357 (0.174, 0.733)** | **0.005** |
| **Declined Vaccine** |  |  |  |  |  |  |  |  |  |  |
| No | 806 | -- | -- | -- | -- | 354 | -- | -- | -- | -- |
| Yes | 164 | 0.654 (0.508,0.841) | 0.001 | **0.659 (0.487, 0.891)** | **0.007** | 42 | 0.359 (0.226,0.571) | <0.001 | **0.306 (0.159, 0.587)** | **<0.001** |
| **Someone in the Household was Vaccinated** | |  |  |  |  |  |  |  |  |  |
| No | 547 | -- | -- | -- | -- | 286 | -- | -- | -- | -- |
| Yes | 406 | 1.908 (1.492,2.439) | <0.001 | 1.953 (1.457, 2.619) | 0 | 105 | -- | -- | -- | -- |
| **Healthworkers as Information Source for Covid-19** | | |  |  |  |  |  |  |  |  |
| No | 546 | -- | -- | -- | -- | 207 | -- | -- | -- | -- |
| Yes | 424 | -- | -- | -- | -- | 189 | 1.446 (1.001,2.088) | 0.049 | 1.095 (0.644, 1.861) | 0.738 |
| **Local Leaders as Information Source for Covid-19** | | |  |  |  |  |  |  |  |  |
| No | 860 | -- | -- | -- | -- | 353 | -- | -- | -- | -- |
| Yes | 110 | 1.507 (1.024,2.215) | 0.037 | 1.574 (0.988, 2.507) | 0.056 | 43 | -- | -- | -- | -- |
| **Trust Scientific Evidence** |  |  |  |  |  |  |  |  |  |  |
| Distrust | 96 | -- | -- | -- | -- | 35 | -- | -- | -- | -- |
| Trust | 771 | 1.608 (1.168,2.213) | 0.004 | 1.089 (0.739, 1.603) | 0.667 | 307 | 1.839 (1.046,3.233) | 0.034 | 1.657 (0.752, 3.649) | 0.21 |
| **Trust in Government Index** |  |  |  |  |  |  |  |  |  |  |
| Low | 523 | -- | -- | -- | -- | 228 | -- | -- | -- | -- |
| High | 441 | 1.474 (1.191,1.823) | <0.001 | 1.05 (0.8, 1.377) | 0.726 | 166 | 1.451 (0.999,2.106) | 0.05 | 0.826 (0.47, 1.454) | 0.508 |
| **Trust WHO** |  |  |  |  |  |  |  |  |  |  |
| Distrust | 105 | -- | -- | -- | -- | 29 | -- | -- | -- | -- |
| Trust | 762 | 2.178 (1.638,2.896) | <0.001 | 1.354 (0.96, 1.91) | 0.084 | 311 | 2.99 (1.748,5.117) | <0.001 | 1.844 (0.823, 4.127) | 0.137 |
| **Ministry of Health Index** |  |  |  |  |  |  |  |  |  |  |
| Low | 214 | -- | -- | -- | -- | 65 | -- | -- | -- | -- |
| High | 723 | 2.088 (1.656,2.633) | <0.001 | **1.713 (1.265, 2.321)** | **0.001** | 314 | 3.768 (2.469,5.75) | <0.001 | **2.337 (1.218, 4.485)** | **0.011** |
| **Truthfulness of Institutions** |  |  |  |  |  |  |  |  |  |  |
| High | 754 | 2.392 (1.896,3.017) | <0.001 | **1.729 (1.269, 2.356)** | **0.001** | 309 | 2.576 (1.722,3.852) | <0.001 | 1.203 (0.598, 2.421) | 0.604 |
| Low | 206 | -- | -- | -- | -- | 77 | -- | -- | -- | -- |

**Supplementary Table 5**

**Association between sex and COVID-19 uptake**

| **Variables** | **Vax** | **No Vax** | **OR (95% CI)** | **OR *p*-value** | **Adjusted OR (95% CI)** | **AOR *p*-value** |
| --- | --- | --- | --- | --- | --- | --- |
| **Age** | | | | | | |
| 18-35 | 932 | 1353 | -- | -- | -- | -- |
| 36-55 | 881 | 780 | 1.87 (1.6,2.19) | <0.001 | **1.68 (1.33,2.11)** | **<0.001** |
| 56-65 | 206 | 117 | 2.91 (2.18,3.9) | <0.001 | **2.07 (1.34,3.22)** | **0.001** |
| 66+ | 142 | 65 | 3.48 (2.43,5) | <0.001 | **5.03 (2.74,9.23)** | **<0.001** |
|  |  |  |  |  |  |  |
|  | | | | | | |
| Male | 1233 | 1308 | -- | -- | -- | -- |
| Female | 928 | 1007 | 1.02 (0.88,1.18) | 0.78 | 1.08 (0.87,1.34) | 0.47 |
|  |  |  |  |  |  |  |
| **Academic Index** | | | | | | |
| Low | 228 | 564 | -- | -- | -- | -- |
| High | 1677 | 1527 | 1.52 (1.24,1.86) | <0.001 | 0.99 (0.74,1.33) | 0.96 |
|  |  |  |  |  |  |  |
| **Assets Index** | | | | | | |
| Low | 687 | 563 | -- | -- | -- | -- |
| Medium | 841 | 940 | 0.82 (0.68,0.99) | 0.04 | **0.71 (0.54,0.95)** | **0.02** |
| High | 613 | 805 | 0.85 (0.7,1.03) | 0.1 | 0.9 (0.66,1.21) | 0.49 |
|  |  |  |  |  |  |  |
| **Education** | | | | | | |
| No School | 110 | 104 | -- | -- | -- | -- |
| Other | 60 | 77 | 1.01 (0.61,1.66) | 0.97 | 0.7 (0.26,1.84) | 0.47 |
| Primary | 441 | 177 | 1.16 (0.8,1.7) | 0.43 | 1.28 (0.59,2.75) | 0.53 |
| Secondary | 806 | 1052 | 0.85 (0.61,1.18) | 0.32 | 1.27 (0.63,2.58) | 0.5 |
| Tertiary/Postgraduate | 710 | 893 | 0.7 (0.51,0.98) | 0.04 | 1.06 (0.52,2.16) | 0.88 |
|  |  |  |  |  |  |  |
| **Employment** | | | | | | |
| Some Employment | 1689 | 1530 | -- | -- | -- | -- |
| Student | 87 | 339 | 0.33 (0.25,0.44) | <0.001 | **0.43 (0.29,0.64)** | **<0.001** |
| Unemployed/Retired/Housewife | 357 | 433 | 0.94 (0.77,1.13) | 0.49 | 0.89 (0.67,1.18) | 0.42 |
|  |  |  |  |  |  |  |
| **Declined Vaccine** | | | | | | |
| No | 1978 | 1860 | -- | -- | -- | -- |
| Yes | 183 | 454 | 0.4 (0.32,0.49) | <0.001 | **0.42 (0.32,0.57)** | **<0.001** |
|  |  |  |  |  |  |  |
| **Heard of Any COVID-19 Vaccines** | | | | | | |
| No | 25 | 200 | -- | -- | -- | -- |
| Yes | 2136 | 2115 | 6.18 (3.95,9.67) | <0.001 | **17.38 (6.97,43.37)** | **<0.001** |
|  |  |  |  |  |  |  |
| **Someone in the Household was Vaccinated** | | | | | | |
| No | 403 | 1371 | -- | -- | -- | -- |
| Yes | 1721 | 904 | 4.04 (3.43,4.76) | <0.001 | **4.51 (3.61,5.65)** | **<0.001** |
|  |  |  |  |  |  |  |
| **Believed Vaccine Distribution was** | | | | | | |
| Unfair | 37 | 40 | -- | -- | -- | -- |
| Fair | 1729 | 1125 | 3.02 (2.51,3.64) | <0.001 | **2.17 (1.71,2.76)** | **<0.001** |
|  |  |  |  |  |  |  |
| **Community as Information Source for COVID-19** | | | | | | |
| No | 1587 | 2009 | -- | -- | -- | -- |
| Yes | 574 | 306 | 1.44 (1.17,1.76) | <0.001 | 1.07 (0.8,1.44) | 0.65 |
|  |  |  |  |  |  |  |
| **Family/friends as Information Source for COVID-19** | | | | | | |
| No | 1670 | 1776 | -- | -- | -- | -- |
| Yes | 491 | 539 | 0.83 (0.71,0.99) | 0.03 | 0.81 (0.64,1.03) | 0.09 |
|  |  |  |  |  |  |  |
| **Healthworkers as Information Source for COVID-19** | | | | | | |
| No | 908 | 1334 | -- | -- | -- | -- |
| Yes | 1253 | 981 | 1.52 (1.31,1.76) | <0.001 | 1.21 (0.98,1.49) | 0.08 |
|  |  |  |  |  |  |  |
| **Local Leaders as Information Source for COVID-19** | | | | | | |
| No | 1636 | 2095 | -- | -- | -- | -- |
| Yes | 525 | 220 | 1.6 (1.28,2.01) | <0.001 | 1.31 (0.96,1.8) | 0.09 |
|  |  |  |  |  |  |  |
| **Phone/Social Media/Internet as Information Source for COVID-19** | | | | | | |
| No | 1168 | 980 | -- | -- | -- | -- |
| Yes | 993 | 1335 | 0.7 (0.6,0.81) | <0.001 | 0.86 (0.68,1.08) | 0.2 |
|  |  |  |  |  |  |  |
| **Tested for COVID-19** | | | | | | |
| No | 2034 | 2242 | -- | -- | -- | -- |
| Yes | 122 | 72 | 2.39 (1.67,3.42) | <0.001 | **2.12 (1.26,3.56)** | **<0.001** |
|  |  |  |  |  |  |  |
| **Scientific Evidence** | | | | | | |
| Distrust | 136 | 260 | -- | -- | -- | -- |
| Trust | 1807 | 1754 | 1.63 (1.26,2.12) | <0.001 | 1.06 (0.74,1.52) | 0.75 |
|  |  |  |  |  |  |  |
| **Trust in Government Index** | | | | | | |
| Low | 1035 | 1306 | -- | -- | -- | -- |
| High | 1087 | 983 | 1.36 (1.18,1.58) | <0.001 | 1.09 (0.88,1.35) | 0.44 |
|  |  |  |  |  |  |  |
| **Trust WHO** | | | | | | |
| Distrust | 153 | 359 | -- | -- | -- | -- |
| Trust | 1740 | 1664 | 1.81 (1.42,2.3) | <0.001 | 1.08 (0.76,1.51) | 0.68 |
|  |  |  |  |  |  |  |
| **Ministry of Health Index** | | | | | | |
| Low | 226 | 611 | -- | -- | -- | -- |
| High | 1855 | 1601 | 2.22 (1.82,2.7) | <0.001 | **1.41 (1.05,1.9)** | **0.02** |
|  |  |  |  |  |  |  |
| **Truthfulness of Institutions Index** | | | | | | |
| Low | 205 | 626 | -- | -- | -- | -- |
| High | 1920 | 1644 | 2.1 (1.73,2.54) | <0.001 | **1.47 (1.09,1.99)** | **0.01** |
